# Preferential sensing and response to microenvironment stiffness of human dermal fibroblast cultured on protein micropatterns fabricated by 3D multiphoton biofabrication

**DOI:** 10.1038/s41598-017-12604-z

**Published:** 2017-09-29

**Authors:** Ming Hui Tong, Nan Huang, Alfonso Hing Wan Ngan, Yanan Du, Barbara Pui Chan

**Affiliations:** 1 Tissue Engineering Laboratory, Department of Mechanical Engineering, The University of Hong Kong, Pokfulam Road, Hong Kong Special Administrative Region, China; 2 Department of Mechanical Engineering, The University of Hong Kong, Pokfulam Road, Hong Kong Special Administrative Region, China; 3 Department of Biomedical Engineering, School of Medicine, Tsing-hua University, Beijing, China

## Abstract

While cells are known to sense and respond to their niche including the matrix and the mechanical microenvironment, whether they preferentially sense and react to the stiffness of their microenvironment regardless of its intrinsic material properties is unknown. In this work, protein micropillar arrays with independently controllable stiffness via alterations in pillar height and elastic modulus via laser power used during photochemical cross-linking, were fabricated using a recently developed multiphoton-based 3D protein micro-patterning technology. Human dermal fibroblasts were cultured on these micropillar arrays and the specific interactions between cells and the protein micropatterns particularly on the formation and maturation of the cell-matrix adhesions, were investigated via immunofluorescence staining of the major molecular markers of the adhesions and the measurement of their cluster size, respectively. Our results showed that the cluster size of focal adhesions increased as the stiffness of the micropillar arrays increased, but it was insensitive to the elastic modulus of the protein micropillars that is one of the intrinsic material properties. This finding provides evidence to the notion that cells preferentially sense and react to the stiffness, but not the elastic modulus of their microenvironment.

## Introduction

A great deal of recent evidence has shown that mechanical microenvironment, particularly elastic modulus and stiffness, plays a very important role in determining the cell fates, such as migration^1,2^, proliferation^3^ and differentiation^4–6^. Cell matrix adhesions are complex structures mediating the interaction and attachment of cells to a substrate and enabling cells to sense such mechanical microenvironment in the extracellular matrix (ECM). The primary sites of cell-matrix interactions are known as matrix adhesions such as focal adhesions^7^ which consist of complex sub-cellular structures, linking the cytoskeleton to the ECM and regulating crucial activities such as angiogenesis^8^ and wound healing^9^. Integrins^10^ are matrix receptors consisting of heterodimers with one α-chain and one β-chain and they initialize the adhesion between cells and their ECM. Different integrins usually have different preferences for different ECM ligands and are involved in different cell behaviors. Both αv and β1 chains of integrin were reported to form the nascent adhesion^11^ and activate FAK family kinase^12^. Paxillin^13^ as another important component of focal adhesions plays an important role in several signaling pathways that regulate cell activities such as attachment^14^ and migration^15^. Upon ECM engagement through integrin receptors, FAK was activated and recruited to the focal adhesions^16,17^. In some studies, the size of the focal adhesion clusters was reported to be positively related to both the elastic modulus^18^ and stiffness^19^ of the ECM.

Although ample evidence has been gathered to show the importance of microenvironment stiffness on cellular fate processes^20,21^, it remains unclear whether the ECM stiffness is an overriding factor, compared with other factors of the ECM such as its intrinsic elastic modulus, which is known to associate with the microstructure of the material. Clarifying this issue is of high significance to the reliability of *in vitro* experimentation, since, firstly, it is never possible to create a microenvironment in the laboratory that can duplicate exactly the *in vivo* conditions. And secondly, one can legitimately query whether experiments performed on different types of microenvironment are directly comparable. If the ECM stiffness can be proven to be a dominating factor for cell fate mechanoregulation, then the need becomes a simple one which is to ensure that the microenvironment used exhibits comparable stiffness between experiments, or with *in vivo* conditions, regardless of its elastic modulus. Both elastic modulus and stiffness measure a substance’s resistance to deformation, but they are not totally the same. Elastic modulus is an intrinsic property of a material that is independent of geometry^22^, while stiffness, measured in Newton per meter, is an extrinsic property of a structure that usually depends on both material and shape conditions^23^. Another reason to differentiate the effects of stiffness and elastic modulus on cellular fates is the confusion of the use of terminologies related to mechanoregulation studies. For example, in a report investigating the dependence of stem cell lineage specification on substrate stiffness^20^, what was being manipulated was actually elastic modulus, measured in Newton per unit area, rather than stiffness. Moreover, some studies used rigidity, instead of stiffness, to denote elastic modulus^21^ but it usually refers to stiffness.

Hydrogel assay has been used to study mechanosensing of stiffness and elastic modulus for long and it has an advantage of allowing for studying both normal and tangential traction stresses. Some studies that made use of polyacrylamide gel as the substrate to culture cells modulated elastic modulus by changing the ratio of substrate to crosslinker concentration ratio^21^. Other studies modulated stiffness by changing the thickness of hydrogel without altering its composition, microtopography and elastic modulus, enabling the decoupling between elastic modulus and stiffness. For tangential deformations and traction stresses, Lin YC. *et al*. showed theoretically and experimentally that altering substrate thickness of polyacrylamide gels modifies the substrate stiffness^24^. For the general 3D case, del Alamo *et al*. developed the theory showing how stiffness depends on substrate thickness^24^. More recently, Aung *et al*. used this idea to show experimentally that cancer cells sense substrate stiffness using matrigel substrates of constant composition but varying thickness^25,26^. However, the differential sensitivity of cells, in terms of cell matrix adhesion maturation, to elastic modulus and stiffness has not been studied.

To study the relative importance of stiffness versus the intrinsic property of the microenvironment on focal adhesion maturation, which is a pre-requisite for important cellular fate processes including proliferation and migration, we employ arecently developed multiphoton-based microfabrication technology^27^ to fabricate micropillar-array substrates for cell culture that exhibits different in-plane stiffness and contact areas sensed by the cells. The technique involved is a 3D printing technology that enables the fabrication of user-defined protein micropatterns with sub-micron resolution and high aspect ratios, allowing spatial and independent control of the topology^27^, porosity^28^, mechanical proprieties^28^ and the type of ECM proteins used, via a wide range of independently controlled fabrication parameters including the scanning power, reagent parameters such as the photosensitizer concentration, and geometry parameters such as the shape of the protein micropatterns. The micropatterns fabricated this way can be used directly as cell culture substrates for cell niche studies particularly on the mechanical and the matrix niche. In this work, we aim to study the maturation of the cell-matrix adhesions of human dermal fibroblasts cultured on protein micropillar arrays with independently controlled stiffness and elastic modulus produced by this fabrication technology.

## Mechanics Foundations

Before discussing the experiments, a clear definition of the stiffness of a substrate for cell culture needs to be given. For a flat substrate without surface patterning, a tangential stiffness may be defined as the magnitude of a force acting tangentially on the substrate surface required to produce unit displacement of the contact region along the force direction. The tangential stiffness of an elastic flat surface over a circular contact region of diameter *D*, as felt by a rigid stylus, is given as^29^

1 $${{\rm{S}}}_{flat}=D(\frac{4{\rm{G}}}{2-\nu })$$

where ν is Poisson ratio and *G* the shear modulus of the surface material. For a homogeneous, flat substrate without elastic anisotropy, all locations on the surface would have the same tangential stiffness defined this way which is also a scalar rather than a tensor.

For a substrate for cell culture with a micropatterned surface, however, the above definition of tangential stiffness is inconvenient as the stiffness value would no longer be location or direction independent. Also, a cell lying on top of a micropatterned substrate may not touch the trough regions of the substrate, and so for the purpose of discussing cell-matrix interactions or mechanoregulation, a more relevant definition of stiffness would be needed. For the type of micropillar-array patterned substrates studied in this work, traction-force microscopy has shown that a cell sitting on such a substrate will exert tangential forces trying to bend each of the contacting micropillars^30^. We therefore define the tangential stiffness of a micropillar array, for the purpose of studying cell-matrix interactions, as the tangential force a rigid cylindrical stylus of diameter *D* would need to exert in order to bend each of the contacting pillars by unit displacement along the force direction. For a single pillar, its bending stiffness is given as

2 $${\rm{s}}=\frac{3{\rm{EI}}}{{{\rm{L}}}^{3}}=\frac{{\rm{3E}}}{{{\rm{L}}}^{{\rm{3}}}}\cdot \frac{\pi {{\rm{d}}}^{{\rm{4}}}}{{\rm{64}}}$$

where *E* is the intrinsic Young modulus of the material, *I* the second moment of the cross-sectional area of the pillar (for a circular pillar), and *L* and *d* are the height and diameter of the pillar respectively. The tangential stiffness of a regular array of micropillars that would be analogous to that in eqn. (1) for a flat substrate is therefore

3 $${{\rm{S}}}_{pillar-array}=(\pi {D}^{2}/4)\cdot n\cdot s$$

where n is the number of pillars per unit area in the array.

In our experiments, micropillar arrays of fixed center-to-center spacing of the pillars were used for cell culture. Thus, in eqn. (3), the areal density *n* of the pillars was a constant, although the pillar geometry and Young modulus were varied to enable *s* to change. Therefore, for a given stylus dimension, i.e. only the bending stiffness of individual pillars matters. The pillar bending stiffness *s* in eqn. (2) is referred simply to as “stiffness” in the following.

In our experiments, we aim to test a hypothesis that cells will preferentially sense and react to the stiffness defined in eqn. (2) above, which is related to both the geometry factors *I* and *L*, as well as the intrinsic Young modulus *E* of the protein material of the micropillars, rather than *E* alone, which is related to the material microstructure or physical nature. As mentioned earlier on, although eqns. (1) to (3) above would make the difference between stiffness and elastic modulus clear in the case of cell-culture substrates, such a difference is often mixed up in the tissue-engineering literature, and hence understanding the specific effects of the two quantities on cell-matrix interactions is important.

## Experimental Results

### Increasing micropillar stiffness increased cell spreading and enhanced focal adhesion maturation

**Figure 1:**
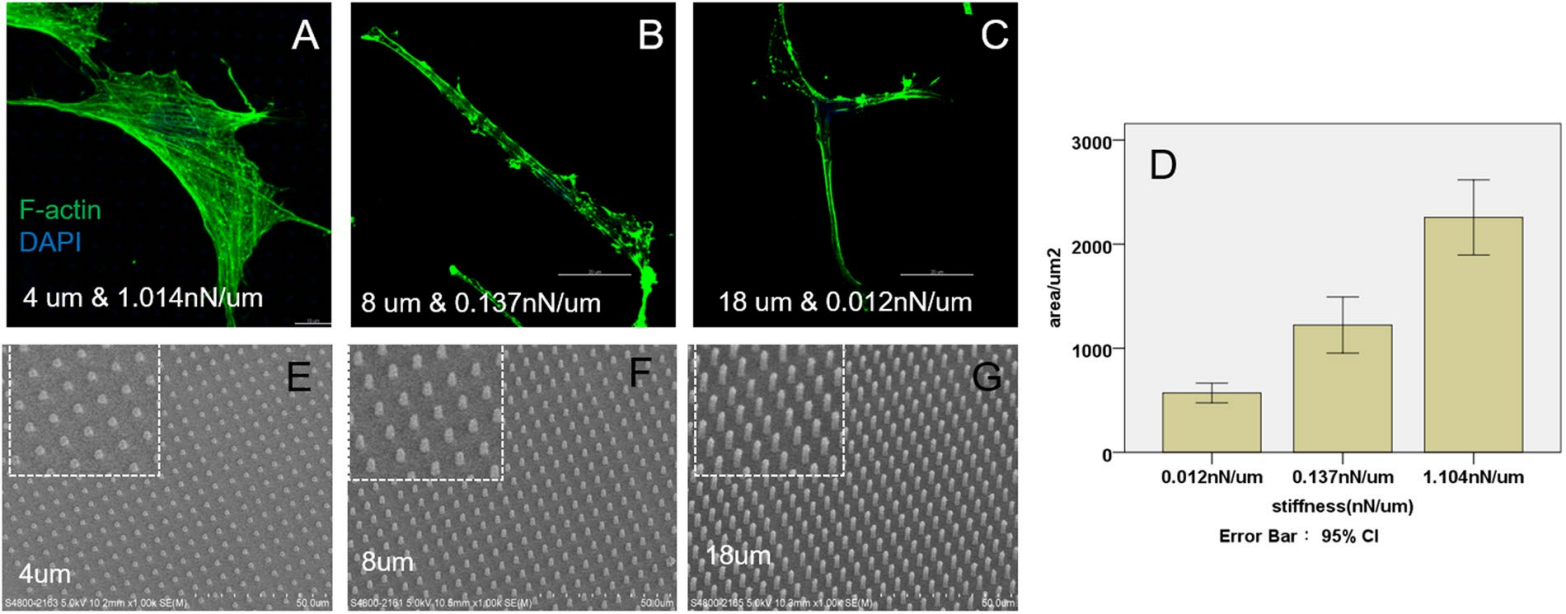
F-actin staining of human dermal fibroblast on micropillars with heights 4 µm (**A**), 8 µm (**B**) and 18 µm (**C**). Scale bar 20 µm. Cell spreading area (**D**) defined as the area of F-actin was quantified by Image J. (n = 10) SEM images were taken of micropillars with heights 4 µm (**E**), 8 µm (**F**) and 18 µm (**G**). Scale bar 50 µm.

According to eqn. (2), keeping the material elastic modulus *E* the same by using the same fabrication parameters for substrate, the stiffness of the micropillars was modulated by varying the height of the micropillars while their diameter and center-to-center spacing were kept at 2 and 6 μm respectively. Figure 1 shows the F-actin expression of human dermal fibroblast after three days’ culture on protein micropillar arrays with different heights and hence stiffness. Cells were widely spread with extensive stress fibers when cultured on arrays of protein micropillars with high stiffness at 1.014 nN/μm, corresponding to a pillar height of 4 µm (Fig. 1A). On micropillars with moderate stiffness of 0.137 nN/μm, corresponding to a micropillar height of 8 µm, cells were less spread and stress fibers less extensive (Fig. 1B). On arrays of protein micropillars with extremely low stiffness at 0.012 nN/µm, corresponding to a micropillar height of 18 µm, cells were least spread with short actin fibers (Fig. 1C). The cell spreading area quantified from the F-actin staining showed an increasing trend as the substrate stiffness increased (Fig. 1D), in that the average cell spread area was <1000 μm^2^ on the softest micropillars (0.012 nN/µm) but >2000 μm^2^ on the stiffest micropillars (1.014 nN/μm). Figure 1E–G shows the SEM images of micropillars with different heights 4 µm (E), 8 µm (F) and 18 µm (G) while, as said before, their diameter and center-to-center spacing are constant at 2 μm and 6 μm respectively.

**Figure 2:**
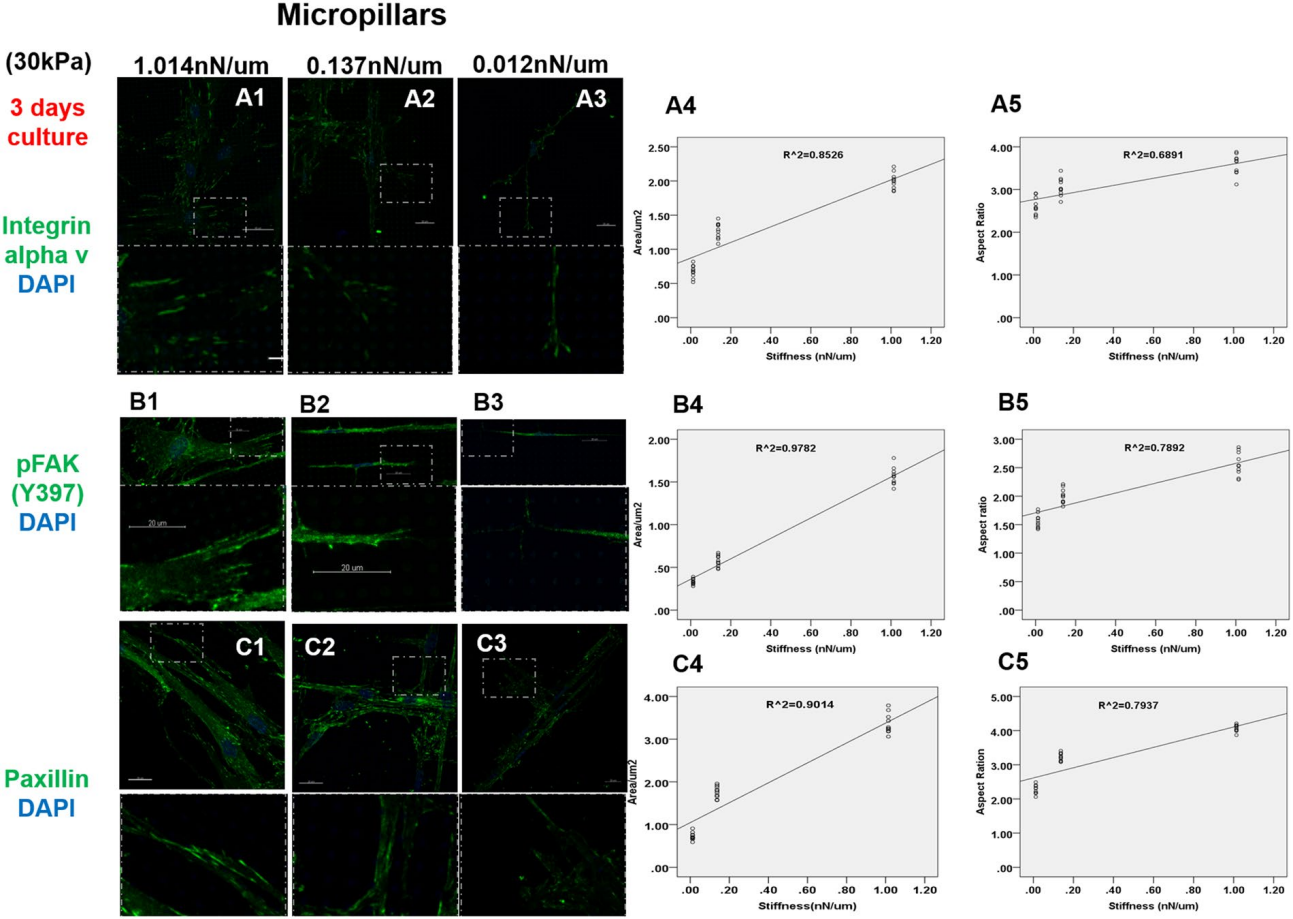
Staining of integrin αv, pFAK(Y397) and paxillin on micropillars with heights 4 µm and stiffness 1.014 nN/µm (**A1 B1 C1**), 8 µm and 0.137 nN/µm (**A2 B2 C2**) and 18 µm and 0.012 nN/µm (**A3 B3 C3**). The expression areas and aspect ratio of integrin αv (**A4–A5**), pFAK(Y397) (**B4–B5**) and paxillin (**C4–C5**) were quantified by Image J. (n = 10) The elastic modulus for all micropillars was 30 kPa. Scale bar 20 µm.

Integrin alpha v, pFAK(Y397) and paxillin are important components of focal adhesions. Figure 2 shows the immunofluorescence staining of these molecular markers for focal adhesions in human dermal fibroblasts after three days’ culture. The size of the focal adhesions on micropillars increased on increasing stiffness, i.e. decreasing height of the micropillars, from small dots in micropillars with height 18 µm (Fig. 2A3) corresponding to a stiffness of 0.012 nN/µm, to small patches on micropillars with 8 and 4 μm heights (Fig. 2A2, A1) corresponding to a stiffness of 0.137 and 1.014 nN/µm, respectively. A statistically significant linear relationship exists between the stiffness of the micropillars and the cluster area of integrin alpha v expression (straight line curve fitting, R^2^ = 0.8526, p < 0.001), between stiffness and the cluster area of pFAK(Y397) expression (straight line curve fitting, R^2^ = 0.9782, p < 0.001) and between stiffness and the cluster area of paxillin expression (straight line curve fitting, R^2^ = 0.9014, p < 0.001). Additionally, another statistically significant linear relationship exists between the stiffness of the micropillars and the aspect ratio of integrin alpha v expression (straight line curve fitting, R^2^ = 0.6891, p < 0.001), between stiffness and the aspect ratio of pFAK(Y397) expression (straight line curve fitting, R^2^ = 0.7892, p < 0.001) and between stiffness and the aspect ratio of paxillin expression (straight line curve fitting, R^2^ = 0.7937, p < 0.001).

### Increasing intrinsic elastic modulus of micropillars did not change focal adhesion maturation

To see whether adhesion maturation is determined by the elastic modulus *E* of the micropillars, micropillar arrays were fabricated with materials of different elastic modulus (15, 30 and 45 kPa) by changing the laser power during fabrication.

**Figure 3:**
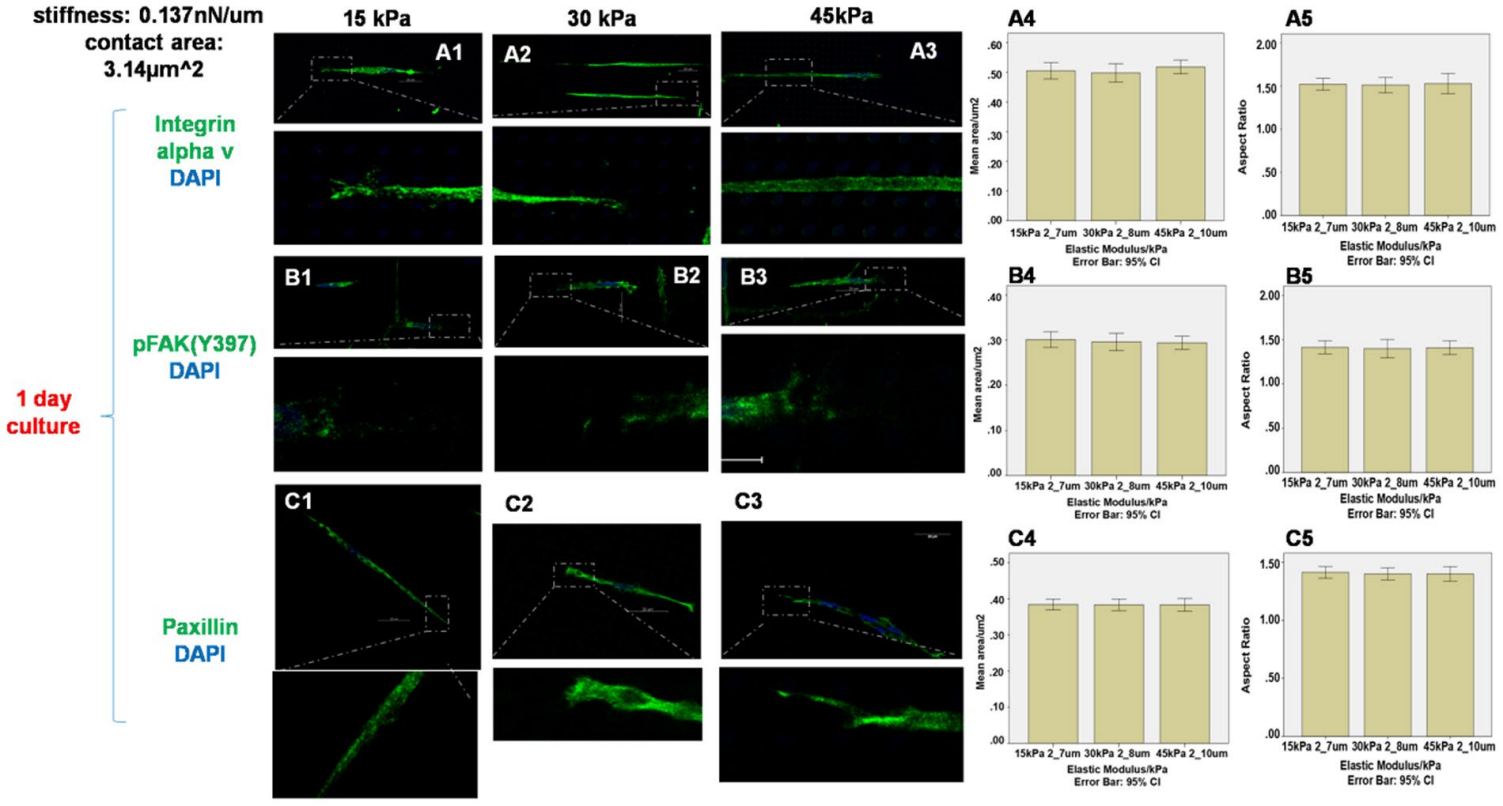
Staining of integrin αv, pFAK(Y397) and paxillin on micropillars with elastic modulus 15 kPa (**A1 B1 C1**), 30 kPa (**A2 B2 C2**) and 45 kPa (**A3 B3 C3**). The expression areas and aspect ratio of integrin αv (**A4–A5**), pFAK(Y397) (**B4–B5**) and paxillin (**C4–C5**) were quantified by Image J. (n = 10) The stiffness and contact area for all micropillars was 0.137 nN/µm and 3.14 μm^2^. Scale bar 20 µm.

**Figure 4:**
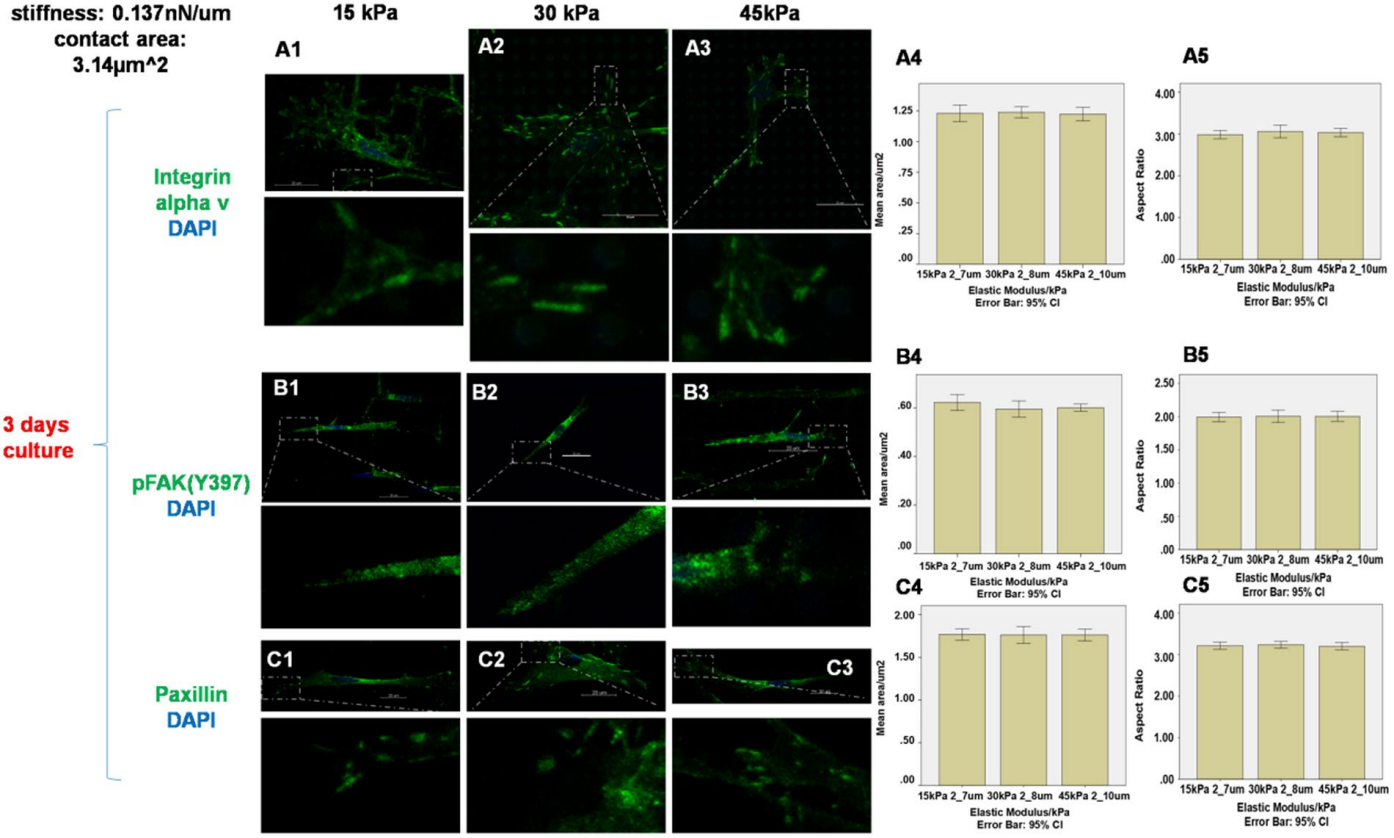
Staining of integrin αv, pFAK(Y397) and paxillin on micropillars with heights 7 µm and elastic modulus 15 kPa (**A1 B1 C1**), 8 µm and 30 kPa (**A2 B2 C2**) and 10 µm and 45 kPa (**A3 B3 C3**). The expression areas and aspect ratio of integrin αv (**A4–A5**), pFAK(Y397) (**B4–B5**) and paxillin (**C4–C5**) were quantified by Image J. (n = 10) The stiffness and contact area for all micropillars was 0.137 nN/µm and 3.14 µm^2^. Scale bar 20 µm.

Figure 3 shows the change in adhesion markers including integrin alpha v, pFAK(Y397) and paxillin at different elastic modulus after 1 day’s culture, while the stiffness of the micropillars was kept constant at 0.137 nN/μm. Increasing the elastic modulus of the micropillars from 15 kPa to 45 kPa, the cluster size of integrin alpha v (Fig. 3A1–A3), pFAK(Y397) (Fig. 3B1–B3) and paxillin (Fig. 3C1–C3) did not change significantly (one-way ANOVA, p = 0.854, 0.800 and 0.994 for integrin alpha v, pFAK(Y397) and paxillin respectively). Additionally, the aspect ratio of integrin alpha v, pFAK(Y397) and paxillin did not change significantly either (one-way ANOVA, p = 0.958, 0.965 and 0.897 for integrin alpha v, pFAK(Y397) and paxillin respectively). The effect at a longer culture duration of 3 days is also the same – Fig. 4 shows that after 3 day' culture, increasing the elastic modulus of the micropillars from 15 kPa to 45 kPa while keeping the stiffness at 0.137 nN/μm did not result in any significant change in both the cluster size and aspect ratio of integrin alpha v (Fig. 4A1–A3), pFAK(Y397) (Fig. 4B1–B3) and paxillin (Fig. 4C1–C3) (one-way ANOVA, p = 0.913, 0.282 and 0.993 for the cluster size and p = 0.590, 0.871 and 0.792 for the aspect ratio of integrin alpha v pFAK(Y397) and paxillin respectively,).

**Figure 5:**
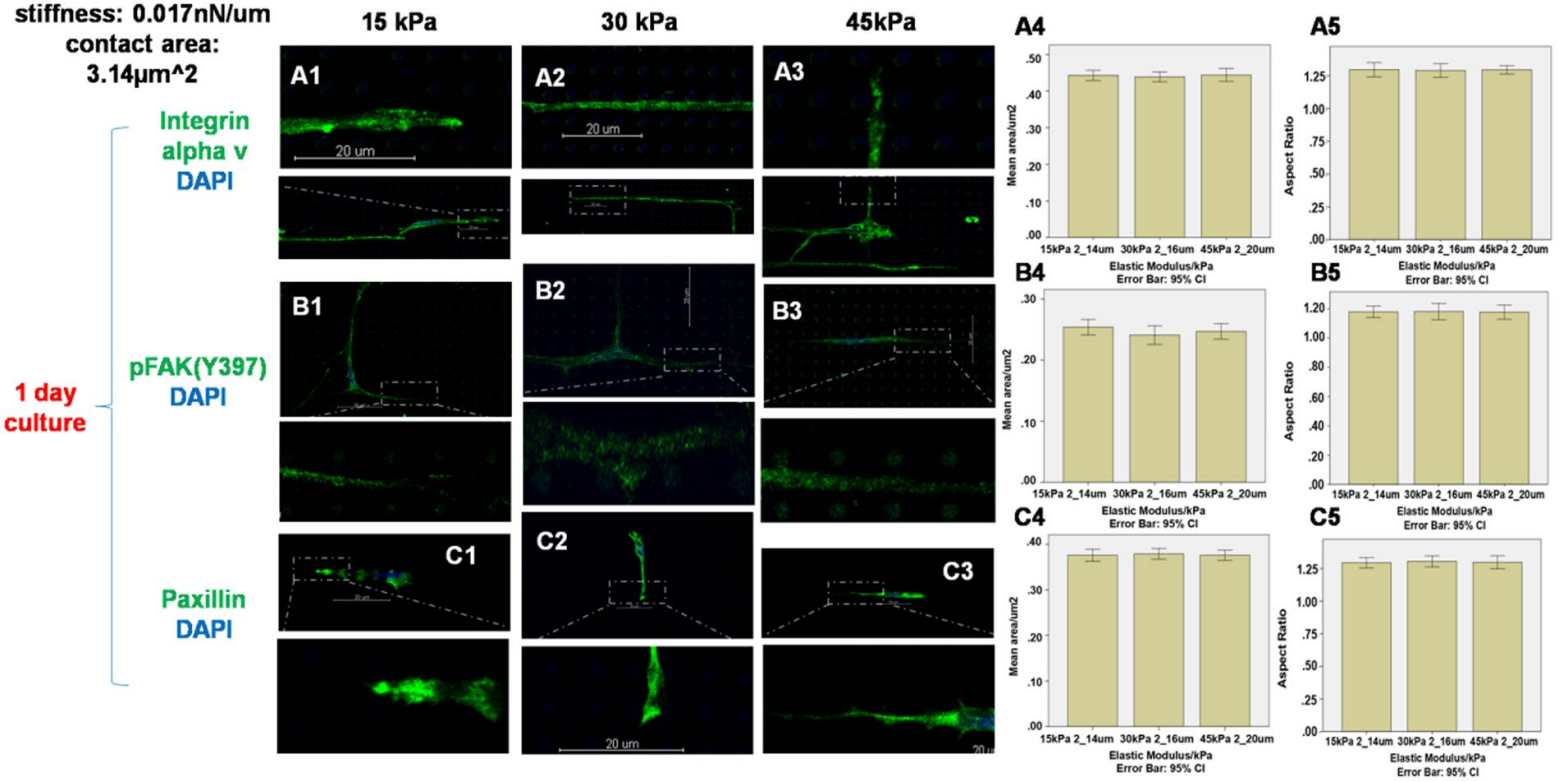
Immunofluorescence staining of integrin αv, pFAK(Y397) and paxillin on micropillars with heights 14 µm and elastic modulus 15 kPa (**A1 B1 C1**), 16 µm and 30 kPa (**A2 B2 C2**) and 20 µm and 45 kPa (**A3 B3 C3**). The expression areas and aspect ratio of integrin αv (**A4–A5**), pFAK(Y397) (**B4–B5**) and paxillin (**C4–C5**) were quantified by Image J. (n = 10) The stiffness and contact area for all micropillars was 0.017 nN/µm and 3.14 μm^2^. Scale bar 20 µm.

In order to study the effect of varying the elastic modulus at different stiffness, apart from a higher stiffness at 0.137 nN/μm shown in Figs 3–4, a lower stiffness of 0.017 nN/μm was also studied. Figure 5 shows the focal adhesion markers of dermal fibroblasts cultured on micropillars of different elastic modulus (15, 30 and 45 kPa) at a low stiffness of 0.017 nN/μm, which was the result of doubling the height of the micropillars (from 7, 8 and 10 μm to 14, 16 and 20 μm for the three elastic moduli). Figure 5A–C show respectively, the integrin alpha v, pFAK(Y397) and paxillin expression on micropillars with the same stiffness 0.017 nN/µm but different elastic modulus of 15 kPa (A1, B1, C1), 30 kPa (A2, B2, C2) and 45 kPa (A3, B3, C3) after one day’s culture. Compared with the expression on micropillars with high stiffness in Fig. 3, weaker expressions of integrin alpha v, pFAK(Y397) and paxillin were observed on all micropillars. Difference in the expressions and aspect ratio of integrin alpha v, pFAK(Y397) and paxillin was not obvious on the micropillars with the same stiffness but different elastic moduli (one-way ANOVA, p = 0.854, 0.325 and 0.901 for the cluster size and p = 0.877, 0.891 and 0.823 for the aspect ratio of integrin alpha v, pFAK(Y397) and paxillin respectively).

**Figure 6:**
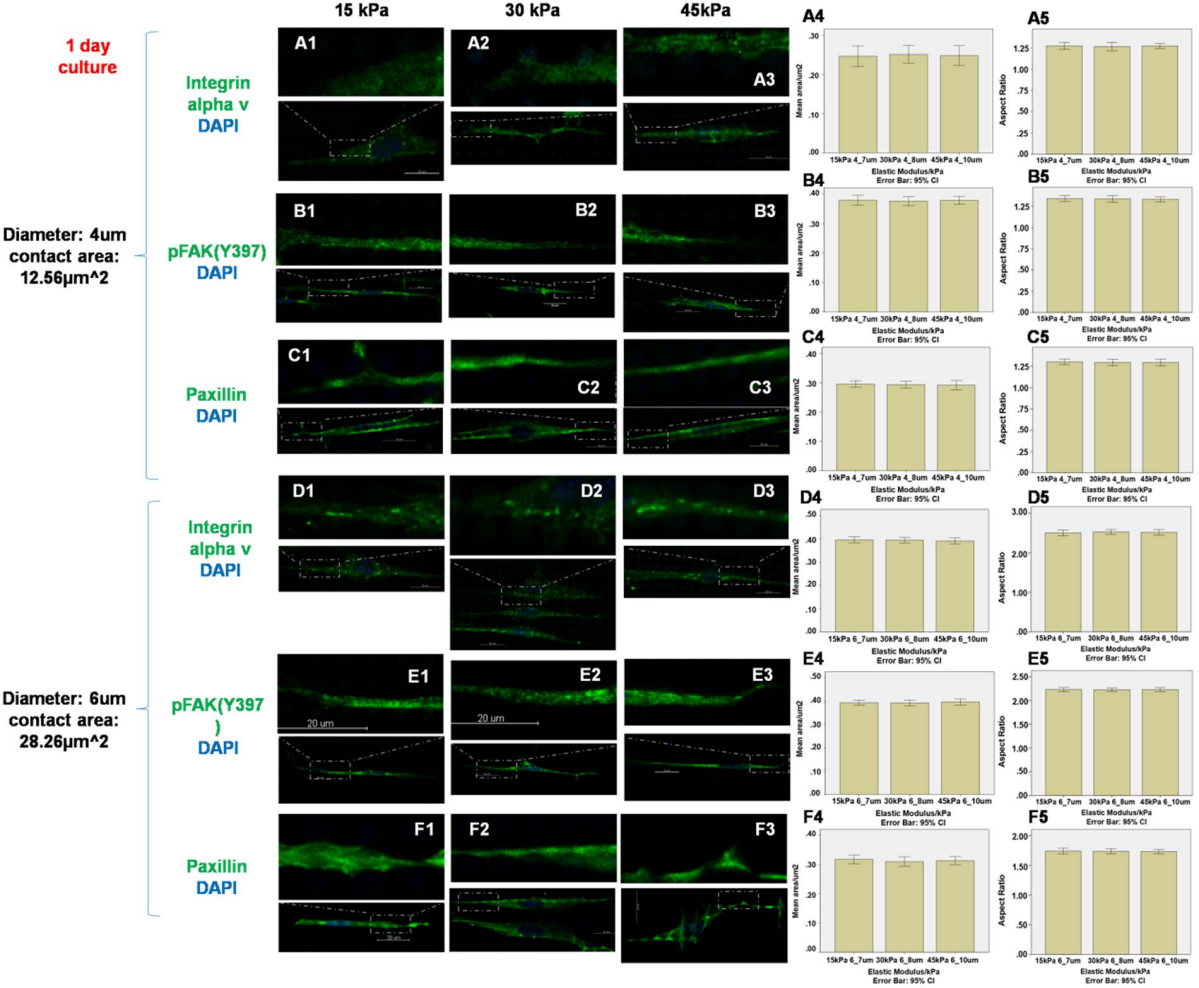
Staining of integrin αv, pFAK(Y397) and paxillin on micropillars with heights 7 µm and elastic modulus 15 kPa (**A1 B1 C1 D1 E1 F1**), 8 µm and 30 kPa (**A2 B2 C2 D2 E2 F2**) and 10 µm and 45 kPa (**A3 B3 C3 D3 E3 F3**). The diameter and contact area of micropillars were 4 µm and 12.56 μm^2^(**A–C**) and 6 µm and 28.26 μm^2^ (**D–F**) respectively. The expression areas and aspect ratio of integrin αv (**A4–A5 D4–D5**), pFAK(Y397) (**B4–B5 E4–E5**) and paxillin (**C4–C5 F4–F5**) were quantified by Image J. (n = 10) Scale bar 20 µm.

To study the effects of varying the elastic modulus against those of the contact area between the cell and each pillar, apart from pillar cross-sectional area of 3.14 μm^2^ used in Figs 3–5, larger pillar areas of 12.56 μm^2^ and 28.26 μm^2^ were also used, by increasing the diameter of the micropillars from 2 to 4 and then to 6 µm, while keeping the culture time (1 day) and micropillar heights (7, 8 and 10 µm) unchanged. Figure 6 shows the integrin alpha v, pFAK(Y397) and paxillin expressions on micropillars with different elastic modulus (15, 30 and 45 kPa) against the contact area factor modulated via micropillar diameter. At each pillar area, expression of integrin alpha v, pFAK(Y397) and paxillin did not demonstrate obvious dependence on the elastic modulus (one-way ANOVA in 12.56 μm^2^ contact area group, p = 0.957, 0.932 and 0.899 for the cluster size and p = 0.929, 0.917 and 0.937 for the aspect ratio of integrin alpha v, pFAK(Y397) and paxillin respectively; one-way ANOVA in 28.26 μm^2^ contact area group, p = 0.854, 0.838 and 0.707 for the cluster size and p = 0.856, 0.972 and 0.955 for the aspect ratio of integrin alpha v, pFAK(Y397) and paxillin respectively).

The results in Figs 3–6 suggest that the cells only sense or react to the stiffness of the micropillar substrate, but not the intrinsic material properties of the micropillars, or the contact area with the micropillar array.

## Discussion

Using the multiphoton-based protein micropillar array platform, this work successfully decoupled two related but distinct mechanical properties of the cell niche, stiffness and elastic modulus. It is found that human dermal fibroblasts are sensitive to the geometry-dependent stiffness, rather than the intrinsic microstructure-dependent elastic modulus, of the protein micropillar arrays in terms of maturation of focal adhesions. These results may contribute to the scaffold design and niche optimization for future applications in 3D cultures and tissue engineering. In addition, this study also provides direct evidence on the differential effects of elastic modulus or stiffness on cellular activities, pointing out the importance to distinguish these two owing to the confusing terminology used in the literature.

### Advantages of the multiphoton protein micropattern system in mechanical niche studies

Previous studies reported that cells cultured on flat substrates such as polyacrylamide gel coated with naturally occurring ECM with varying elastic modulus commit into different lineages^6^ and reveal different preferences of spreading^31^ and proliferation^3,32^. However, these two parameters cannot be decoupled in most studies and the question on whether cell differentially sense these two related but distinct properties cannot be studied, despite that some recent studies of hydrogel assay modulate stiffness by changing the thickness of hydrogels, in which stiffness is decoupled from elastic modulus successfully^25,26^. Micropatterns with custom-designed structural features such as the PDMS-based micropost system^19,33^ and our multiphoton-based protein micropillar array system^27,28^ can be used to decouple different mechanical properties by varying the structural parameters such as height and diameter of the microposts or micropillars for stiffness control and varying the fabrication parameters such as material concentration, power and time during fabrication. However, PDMS-based system is inert to cells that ECM coating is necessary to initiate cell-substrate interactions including adhesions. Many studies in PDMS-based system used microcontact printing^34–36^ of fibronectin^37,38^ or collagen^39–41^ to enable cell adhesion to the system. Coating ECM proteins will artificially increase the integrin clustering and hence made cells on soft substrates behave like on rigid ones^42^. As a result, in PDMS-based system, cell activities cannot be studied without ECM coating because adhesion is a pre-requisite of subsequent activities including proliferation, migration and differentiation, while if ECM is coated, the differential effects of stiffness and elastic modulus on cell matrix adhesion can only be studied with the effects from the coated ECM. In this regard, our multiphoton-based protein micropillar array system is ideal to study the differential sensitivities of cells towards the two related but distinct mechanical properties, stiffness and elastic modulus, without the interference from the coated ECM. This is because our protein micropillar system is cytocompatible and cells can bind without the need to coat specific ECM components^27^, even though the ECM can be separately incorporated and controlled using separate process parameters. Distinguishing the differential sensitivities of cells towards stiffness and elastic modulus in cell niche studies has profound effects and implications on the design of scaffolds for future applications in 3D cultures and tissue engineering, particularly on the structural features, the hierarchical organization and the choice of materials. The current study, utilizing a multiphoton-based protein micropatterning technology, studied the maturation of cell matrix adhesions in human dermal fibroblasts in response to protein micropillar arrays with different stiffness or elastic modulus, after decoupling these two related and distinct parameters.

### Human fibroblasts prefer to sense stiffness but not the intrinsic property elastic modulus

Stiffness, the rigidity of an object, measures the resistance of deformation in response to an applied force and it can be varied by both elastic modulus and shape factors, such as height and diameter^23^. Our multiphoton-based 3D micropatterning technology enables independent control of different mechanical properties^28^. We manipulate the stiffness of the micropillars by varying their height, similar to that of the PDMS microposts^19^. Upon cell binding to a specific substrate, formation of focal adhesions consisting of integrin alpha v, paxillin and the signaling molecule pFAK(Y397) is a sensitive measure of the intimate cell-matrix interaction, which is a pre-requisite to many cellular fate processes including proliferation and migration. Cell on rigid substrates expressed large focal adhesion compared with small adhesion on compliant surface^43^, suggesting that integrin clustering and adhesion size are sensitive markers for cell-matrix interactions. Adhesion maturation occurred with assembly of stress fiber and enhanced size of complex adhesion components. In our study, significant differences in the expression pattern of F-actin and cluster size of integrin alpha v, pFAK(Y397) and paxillin were observed among different stiffness groups. Specifically, on the shortest and most stiff micropillars, cells formed firm adhesions as indicated by large clusters of integrin alpha v, pFAK(Y397) and paxillin. While on the longest and most compliant micropillars, loose adhesions, as indicated by the relatively small integrin alpha v pFAK(Y397) and paxillin clusters were formed. These results suggest that dermal fibroblasts developed more mature adhesions on stiffer and shorter pillars, corroborating with a previous study using human mesenchymal stem cells on PDMS microposts^19^. Our micropillar platform could achieve large range of stiffness, at about 1000 folds of difference (~0.012 to 10 nN/µm) while the PDMS micropost system also has a ~1000 folds of difference in stiffness (at a high range from 1.90 to 1556 nN/µm), mainly achieved through change in the aspect ratio or height of the micropillars or microposts. However, our protein micropillar system is more biomimetic with much lower stiffness mainly results from the lower intrinsic material property mimicking that of the native tissues, at an elastic modulus of 30 kPa in our micropillars platform, in contrast to 3 MPa in the PDMS microposts. The cellular activity of focal adhesion maturation suggests that cells are sensitive to the geometry dependent stiffness. Our results in the second part of the study also showed that the cell activity in adhesion maturation is not attributable to the intrinsic property elastic modulus of the micropillars, suggesting that the geometrical features are what the cells sense. Quantifications of expression areas of integrin alpha v, pFAK(Y397) and paxillin clusters demonstrated no obvious differences among groups with three elastic moduli, ranging from 15 to 45 kPa, made no difference on adhesion size and hence maturation. This is apparently contradictory to previous findings[2] that polyacrylamidegel with low elastic modulus (E~1 kPa) resulted in diffuse and dynamic adhesions while the gel with high elastic modulus (E~30 to 100 kPa) promote stable adhesions. Firstly, results of the current study may not be directly compared to that study because the substrate systems used were different. Specifically, a flat polyacrylamide (PA) gel was used as the substrate in that study while a protein micropillar array was used in the current study. The local stiffness of the polyacrylamide gel system changed as the elastic modulus was varying through changing the ratio of acrylamide:bis-acrylamide and therefore the compliant gels with low modulus (E~1 kPa) was also having a low stiffness. Since the PA gel system in that study did not decouple stiffness from elastic modulus, any difference in cellular activities might be resulted from the local stiffness, but not the elastic modulus. While in our micropillar array system, we decoupled stiffness and elastic modulus through a different set of fabrication parameters and found that cells are more sensitive to the stiffness but not the elastic modulus of the micropillar arrays, even against other factors including culture time, stiffness and contact area. A recent study[43] investigated the effects of elastic modulus on focal adhesion maturation using both polyacrylamide gel (flat gel) and PDMS (microposts) platforms, in the presence of collagen coating. Interestingly, the dependence of focal adhesion maturation on the elastic modulus was only found in the polyacrylamide gel (flat gel) platform but not the PDMS microposts substrate with elastic modulus ranging from 0.1 kPa to 2.3 MPa. The study attributed the finding to the difference in ECM protein tethering between the polyacrylamide gel and the PDMS systems. In other words, the ECM protein coating distributed evenly on all PDMS substrates ranging from 0.1 kPa to 2.3 MPa while the amount of collagen coating on polyacrylamide gel was positively related to the elastic modulus. This study concludes that it was the amount and distribution of collagen that dominated the different cellular activities. Our albumin micropillar system shows that varying the elastic modulus of the micropillars did not stimulate focal adhesion maturation, corroborating with the results from this PDMS micropost system. Although our current study did not use a flat gel system, we did vary the contact area of the micropillar system within a narrow range between 2 to 6 µm in diameter while varying the elastic modulus of the micropillars and found no difference in adhesion maturation. This increase in contact area of the micropillar system is different from the flat gel system as a fibroblast still sees a micropillar with a contact area of 6 µm as a pillar while the PA flat gel is much larger than a single cell. Our separate study on decoupling the matrix niche and the mechanical niche did corroborate with the result on sensitivity towards elastic modulus in a flat gel system with an area of 100 × 100 µm. Our micropillar system can decouple the matrix niche from the mechanical niche, a verifying example is the differential traction force generation response from fibroblasts on micropillar arrays with and without fibronectin matrix coating as shown in the Supplementary data in our previous report^28^. Moreover, the range of elastic modulus studied in these two studies were different. The elastic moduli of the protein micropillars in the current study were comparable to the moderate to high elastic modulus used in that study but the PA gels with low elastic modulus at ~1 kPa was outside the range of elastic modulus achieved by the micropillar system. The range of elastic modulus used in the protein micropillar array system was around 10-60 kPa^28^, different from that of the PA gel system, ranging from 0.1 kPa to 40 kPa, particularly at the lower bound^6^. It was therefore important to further expand the range of elastic modulus achieved by the multiphoton-based protein micropillar system. Increasing the elastic modulus above 45 kPa by changing the fabrication parameters will easily lead to burning while decreasing the elastic modulus below 15 kPa will easily result in collapse of the micropillars particularly in those with high aspect ratio. Using a cooling system to reduce the chance of burning, changing to protein materials other than bovine albumin and photosensitizer other than rose Bengal, may further expand the range of the elastic modulus achievable by the current micropillar system.

## Methods

### Two-photon photochemical crosslinking system

A two-photon confocal laser scanning microscopy system (Zeiss 710, Carl-Zeiss, GmbH, Jena), equipped with a mode-locked Ti:Sapphire femtosecond near infrared (NIR) laser (Coherent, Inc., Santa Clara, California, USA) was used to fabricate the protein microstructures. The laser was tuned to 800 nm and a 40x/ 1.3 N.A. oil-immersion objective lens was used to aid the multiphoton fabrication process. The laser power was measured using a power meter (Coherent, Inc.) before each round of fabrication.

### Sample loading and autofocusing

Glass-bottomed 35 mm confocal culture dishes (P35G-1.5-10-C, MatTek Corp., Ashland, MA, USA) were used as the substrate for fabrication. Each confocal dish was sterilized under UV light for 15 min before use. Aliquots of BSA solution (50 µl) (Bovine Serum Albumin, Sigma-Aldrich Corp., St Louis, MO, USA) and the photosensitizer, rose Bengal (RB; Sigma-Aldrich) were mixed at pre-determined ratios and then loaded into the center of the confocal culture dish. The dish was mounted onto the stage of the inverted Zeiss 710 microscope, after which the autofocus function in the Z-710 reflection mode was used in conjunction with an Argon laser at 488 nm excitation to find the liquid-solid interface between the protein solution and the glass bottom of the culture dish. The interface was marked as the zero position in the z-direction.

### Preparation of the reagents and fabrication of BSA micropillars and matrices

Stock solutions of RB (at 2% w/v in 1xPBS) and BSA (at 367 mg/ml in 1xPBS) were freshly prepared prior to each experiment. Both solutions were mixed at a ratio of 1:9 and final concentration prepared for fabrication is RB (0.2% w/v) and BSA (330 mg/ml). Protein micropillars that were cylindrical in shape were fabricated by using the region of interest (ROI) function of the microscope software. Micropillar diameter was kept constant at 2 µm except the contact area study in which the micropillar diameter was changed to 4 µm and 6 µm. Edge-to edge spacing between two adjacent micropillars was always 4 µm. The scanning start position was set at 1 µm below the interface to make sure that microstructures were firmly attached to the bottom of the dish, whereas the scanning end position was set above the interface. The fabrication parameters were set as follows: scanning speed = 1.27 µs/pixel, number = 1, cycle = 1, z-stack interval = 0.5 µm.

### Measurement of bending stiffness of micropillars

The bending stiffness of the protein micropillars can be controlled by varying their height and diameter. The bending stiffness of the single micropillar was calculated using eqn. (2) in the main text. Elastic modulus *E* was measured using a rate jump method^44^ while the fabrication parameter scanning power was used to modulate the elastic modulus due to its strong linear relationship with elastic modulus. Therefore the stiffness can be modulated by changing the elastic modulus, the height or the diameter of micropillars. Setting diameter at 2 µm, micropillars with an elastic modulus of 15 kPa, 30 kPa and 45 kPa at a height of 7 µm, 8 µm and 10 µm respectively, have the same stiffness of 0.137 nN/µm. When all the heights were doubled, that was 14 µm, 16 µm and 20 µm, the stiffness was changed to 0.017 nN/µm.

### Cell culture and reagents

Human dermal fibroblasts(hDFs) from neotissues were obtained from commercial source (CC-2509 NHDF-Neo, Lonza, Basel, Switzerland) and cultured in growth medium containing low-glucose Dulbecco’s modified Eagle’s medium (DMEM, Invitrogen, Grand Island, NY), 10% fetal bovine serum (FBS, Invitrogen), and 100 μg/ml penicillin and 100 μg/ml streptomycin (pen/strep) (Invitrogen). Cells were seeded on BSA micropillars with a density of 1E5/ml, and cultured for 1 or 3 days prior to the immunofluorescence staining. No extracellular matrix proteins were coated on the BSA micropillars.

### Immunofluorescence staining

Focal adhesion markers including F-actin, integrin αv, integrin β1 and paxillin were visualized by either fluorescence staining (F-actin) or immunofluorescence staining (other proteins). In the immunofluorescence staining process, samples were fixed with 4% paraformaldehyde for 10 min at room temperature and then washed with 1xPBS for 3 × 5 min. Cells were then permeabilized with0.5%Tween 20 for 10 min, after which the samples were washed again with 1xPBS for 3 × 5 min. Samples were blocked in 5% BSA in PBS for 30 min and then respectively incubated with a primary monoclonal anti-β-actin antibody (1:100, A2228, Sigma-Aldrich), anti-integrin αv antibody (1:200, ab16821, Abcam), anti-integrin β1 antibody (1:200, ab30394, Abcam), anti-paxillin antibody (1:200, ab32084, Abcam) and anti-pFAK(Y397) antibody (1:200, ab39967, Abcam) at 4 °C overnight. They were then washed three times with 1xPBS, after which they were incubated with secondary antibody (1:400, Invitrogen) for one hour. Samples were then washed three times with 1xPBS and mounted in Fluro-gel II (EMS) containing DAPI. Z-stack images were taken at 0.5 µm intervals using the Zeiss LSM710 confocal system with a 63x/1.4 N.A. objective lens (Plan-Apochromat) and analyzed with the 3D image reconstruction software Imaris (Bitplane, Zurich, Switzerland).

### Image analysis

**Figure 7:**
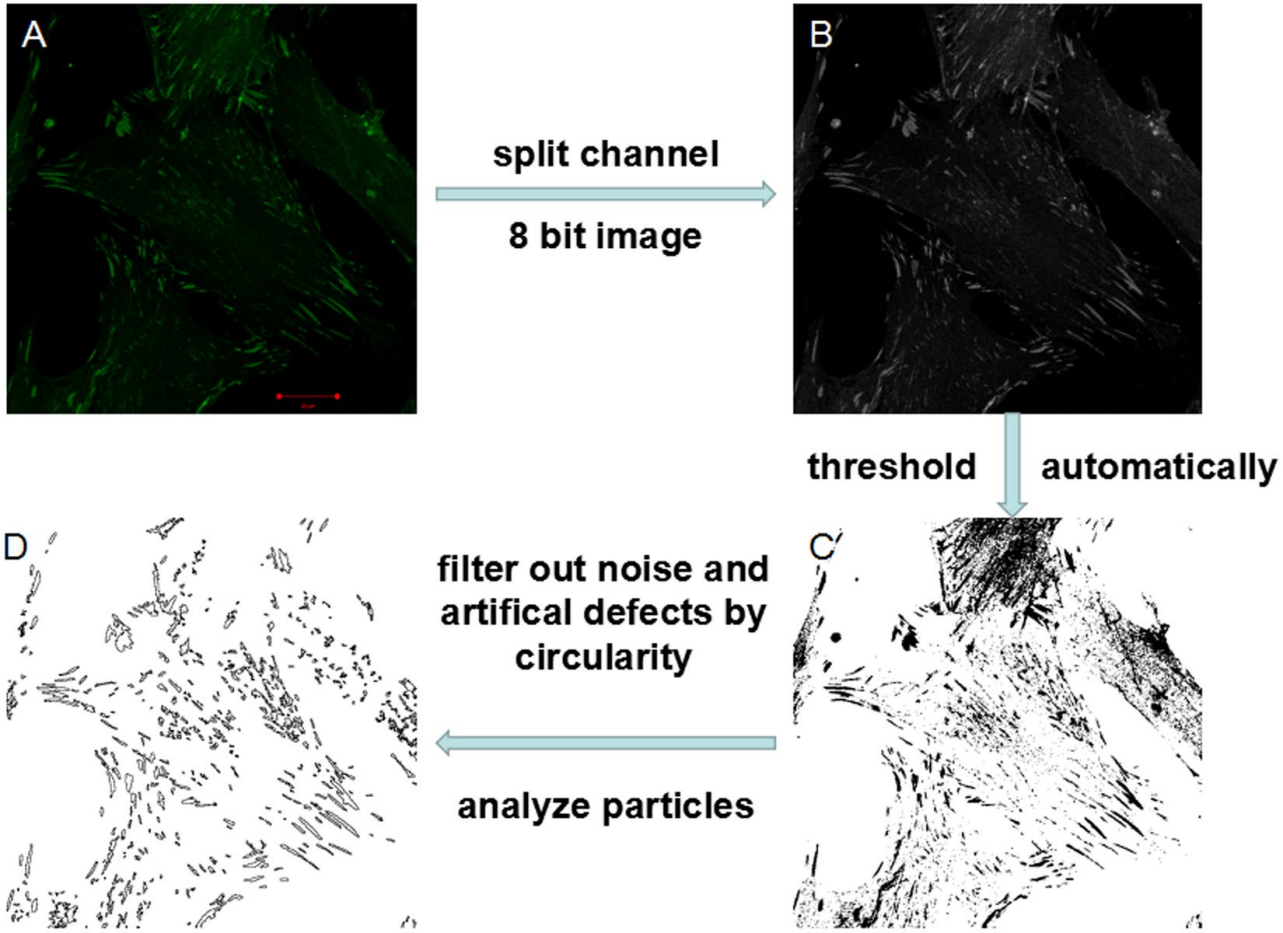
The process of cluster size quantification. An taken image (**A**) was loaded to Image J and transformed to an 8-bit image (**B**). The threshold set automatically was applied (**C**) and then the function of ‘analyze particles’ was used to quantify and summarize the statistics on the size of clusters with circularity between 0.1 and 0.9 (Fig. 7D).

Software Image J was used for the measurement of cluster size. An image (Fig. 7A) which contains single cell was chosen and loaded into Image J. After loading, it was transformed to an 8-bit image (Fig. 7B) for thresholding (Fig. 7C). The threshold set automatically by Image J was applied to the image and some artifacts with extremely high intensity were filtered out. The function of ‘analyze particles’ was then used to quantify and summarize the statistics on the size of clusters with circularity between 0.1 and 0.9 (Fig. 7D). Clusters with circularity below 0.1 and above 0.9 were treated as noises. For all analyses, at least ten cells were randomly chosen and analyzed.

### Statistical analysis

Quantitative information regarding the cluster size of integrin alpha v, integrin beta 1, pFAK (Y397) and paxillin as well as the cell spreading area are presented as mean ± SEM. The normality assumption was verified with the Kolmogorov-Smirnov test and the equal variance assumption was verified by Levene’s test to justify the use of parametric tests. Linear curve fitting was conducted to determine the association between the stiffness and the focal adhesion markers including integrin alpha v, pFAK(Y397) and paxillin. The coefficient of determination (R^2^) was reported in all association studies. One-way ANOVA (with the appropriate post-hoc tests) was used to reveal the differences in integrin alpha v, pFAK(Y397) and paxillin cluster size when culturing cells on protein micropillar arrays of same stiffness but different elastic modulus. For data with equal variance assumed, Bonferroni’s test was used. For data without equal variances, Dunnett’s T3 test was used. SPSS 23.0 (Armonk, NY) and OriginPro 9 (OriginLab, Northampton, MA) were used to execute all analyses and the statistical significance was set at α = 0.05.

### Data availability statement

All data generated or analyzed during this study are included in this published article.
